# Supplementary material for: Machine Learning Accelerates Crystallization for Structure Determination
Source: Angew Chem Int Ed Engl. 2026 May 4;65(25):e1218503. doi: 10.1002/anie.1218503 (PMC13266958; doi:10.1002/anie.1218503)

checkCIF/PLATON report

Structure factors have been supplied for datablock(s) lcz073

THIS REPORT IS FOR GUIDANCE ONLY. IF USED AS PART OF A REVIEW PROCEDURE FOR PUBLICATION, IT SHOULD NOT REPLACE THE EXPERTISE OF AN EXPERIENCED CRYSTALLOGRAPHIC REFEREE.

No syntax errors found. CIF dictionary Interpreting this report

Datablock: lcz073

Bond precision:	C-C = 0.0107 A		Wavelength=1.54184
Cell:	a=12.8671(1)	b=12.0958(2)	c=23.4124(3)
	alpha=90	beta=90.779(1)	gamma=90
Temperature:	100 K		

	Calculated	Reported
Volume	3643.52(8)	3643.52(8)
Space group	P 21/n	P 1 21/n 1
Hall group	-P 2yn	-P 2yn
Moiety formula	C30 H14 Ag3 Cl F18 N6 O	C15 H3 Ag3 F18 N6, C15 H11 Cl O
Sum formula	C30 H14 Ag3 Cl F18 N6 O	C30 H14 Ag3 Cl F18 N6 O
Mr	1175.53	1175.53
Dx,g cm-3	2.143	2.143
Z	4	4
Mu (mm-1)	14.695	14.695
F000	2256.0	2256.0
F000’	2267.87	
h,k,lmax	16,15,29	16,14,29
Nref	7826	7501
Tmin,Tmax	0.156,0.128	0.397,1.000
Tmin’	0.043	

Correction method= # Reported T Limits: Tmin=0.397 Tmax=1.000
AbsCorr = MULTI-SCAN

```
Data completeness= 0.958           Theta(max)= 78.252

R(reflections)= 0.0558( 6387)       wR2(reflections)= 0.1359( 7501)

S = 1.087                Npar= 560
```

```
The following ALERTS were generated. Each ALERT has the format
       test-name_ALERT_alert-type_alert-level.
Click on the hyperlinks for more details of the test.
```

Alert level C

```
PLAT042_ALERT_1_C Calc. and Reported MoietyFormula Strings  Differ     Please Check
              Calc: C30 H14 Ag3 Cl F18 N6 O
              Rep.: C15 H3 Ag3 F18 N6, C15 H11 Cl O
PLAT213_ALERT_2_C Atom F00H            has ADP max/min Ratio .....        3.1 prolat
PLAT213_ALERT_2_C Atom F012            has ADP max/min Ratio .....        3.1 prolat
PLAT213_ALERT_2_C Atom F017            has ADP max/min Ratio .....        3.6 prolat
PLAT213_ALERT_2_C Atom F01N            has ADP max/min Ratio .....        3.1 prolat
PLAT342_ALERT_3_C Low Bond Precision on  C-C Bonds ..............    0.01071 Ang.
PLAT906_ALERT_3_C Large K Value in the Analysis of Variance ......      7.027 Check
PLAT906_ALERT_3_C Large K Value in the Analysis of Variance ......      2.228 Check
PLAT911_ALERT_3_C Missing FCF Refl Between Thmin & STh/L=    0.600         19 Report
                0  2  0, -13  0  1,  -4  0  2,  -3 14  2,  -6 13  4,   3 14  4,
               10 10  6,  -4 12  7,  -3 11  8,  -7 12  9,  -7 11 10,  -3  4 10,
               -6 11 11, -11  8 12,   1 12 15,   2 12 15,   2 10 19,   1 10 20,
                0  0 28,
```

Alert level G

```
PLAT002_ALERT_2_G Number of Distance or Angle Restraints on AtSite          7 Note
PLAT003_ALERT_2_G Number of Uiso or U(i,j)  Restrained non-H-Atoms          6 Report
PLAT004_ALERT_5_G Polymeric Structure Found with Maximum Dimension          1 Info
PLAT083_ALERT_2_G SHELXL Second Parameter in WGHT  Unusually Large      40.69 Why ?
PLAT176_ALERT_4_G The CIF-Embedded .res File Contains SADI Records          3 Report
PLAT186_ALERT_4_G The CIF-Embedded .res File Contains ISOR Records          3 Report
PLAT242_ALERT_2_G Low    'MainMol' Ueq as Compared to Neighbors of       C00X Check
PLAT242_ALERT_2_G Low    'MainMol' Ueq as Compared to Neighbors of       C011 Check
PLAT242_ALERT_2_G Low    'MainMol' Ueq as Compared to Neighbors of       C01E Check
PLAT242_ALERT_2_G Low    'MainMol' Ueq as Compared to Neighbors of       C01G Check
PLAT242_ALERT_2_G Low    'MainMol' Ueq as Compared to Neighbors of       C01L Check
PLAT242_ALERT_2_G Low    'MainMol' Ueq as Compared to Neighbors of       C01N Check
PLAT301_ALERT_3_G Main Residue  Disorder ..............(Resd    1)         5% Note
PLAT720_ALERT_4_G Number of Unusual/Non-Standard Labels ..........         73 Note
              Ag01    Ag02    Ag03    Cl04    F005    F006    F007    F009
              F00B    O00C    F00E    N00F    N00G    F00H    F00I    F00J
              N00K    F00L    F00M    F00N    C00O    C00P    H00P    C00Q
              C00R    C00S    H00S    C00T    H00T    C00U    C00V    C00W
              H00W    C00X    C00Y    H00Y    C00Z    C010    C011    F012
              C013    C014    H014    F015    F016    F017    C018    H018
              C019    H019    C01A    H01A    C01B    C01C    F01D    C01E
              C01F    H01F    C01G    C01H    H01H    C01I    H01I    C01J
              H01J    C01K    H01K    C01L    F01M    C01N    F01N    F0
              F01E
```

```
PLAT860_ALERT_3_G Number of Least-Squares Restraints ............         39 Note
PLAT883_ALERT_1_G Absent Datum for _atom_sites_solution_primary ..     Please Do !
PLAT910_ALERT_3_G Missing FCF Reflection(s) Below Theta(Min)[Deg]=       4.11 Note
               -1  0  1,   1  0  1,   0  0  2,
PLAT912_ALERT_4_G Missing # of FCF Reflections Above STh/L=  0.600        266 Note
PLAT913_ALERT_3_G Missing # of Very Strong Reflections in FCF ....          2 Note
                0  2  0,  -4  0  2,
PLAT933_ALERT_2_G Number of HKL-OMIT Records in Embedded .res File          7 Note
                3 14  6,   5 13  9,   3 14  9,   3 14  8,   3 14  7,   3 14 10,
                3 14  4,
PLAT941_ALERT_3_G Average HKL Measurement Multiplicity ..........        3.1 Low
PLAT969_ALERT_5_G The 'Henn et al.' R-Factor-gap value ..........      3.552 Note
              Predicted wR2: Based on SigI**2  3.83 or SHELX Weight 12.50
PLAT978_ALERT_2_G Number C-C Bonds with Positive Residual Density.          0 Info
```

```
   0 ALERT level A = Most likely a serious problem - resolve or explain
   0 ALERT level B = A potentially serious problem, consider carefully
   9 ALERT level C = Check. Ensure it is not caused by an omission or oversight
  23 ALERT level G = General information/check it is not something unexpected

   2 ALERT type 1 CIF construction/syntax error, inconsistent or missing data
  15 ALERT type 2 Indicator that the structure model may be wrong or deficient
   9 ALERT type 3 Indicator that the structure quality may be low
   4 ALERT type 4 Improvement, methodology, query or suggestion
   2 ALERT type 5 Informative message, check
```

Validation response form

Please find below a validation response form (VRF) that can be filled in and pasted into your CIF.

```
# start Validation Reply Form
_vrf_PLAT042_lcz073
;
PROBLEM: Calc. and Reported MoietyFormula Strings  Differ     Please Check
RESPONSE: ...
;
_vrf_PLAT213_lcz073
;
PROBLEM: Atom F00H            has ADP max/min Ratio .....        3.1 prolat
RESPONSE: ...
;
_vrf_PLAT342_lcz073
;
PROBLEM: Low Bond Precision on  C-C Bonds ..............    0.01071 Ang.
RESPONSE: ...
;
_vrf_PLAT906_lcz073
;
PROBLEM: Large K Value in the Analysis of Variance ......      7.027 Check
RESPONSE: ...
;
_vrf_PLAT911_lcz073
;
```

```
PROBLEM: Missing FCF Refl Between Thmin & STh/L=    0.600         19 Report
RESPONSE: ...
;
# end Validation Reply Form
```

It is advisable to attempt to resolve as many as possible of the alerts in all categories. Often the minor alerts point to easily fixed oversights, errors and omissions in your CIF or refinement strategy, so attention to these fine details can be worthwhile. It is up to the individual to critically assess their own results and, if necessary, seek expert advice.

PLATON version of 17/09/2025; check.def file version of 17/09/2025

---

duplicate check

No duplication found

Datablock lcz073 - ellipsoid plot

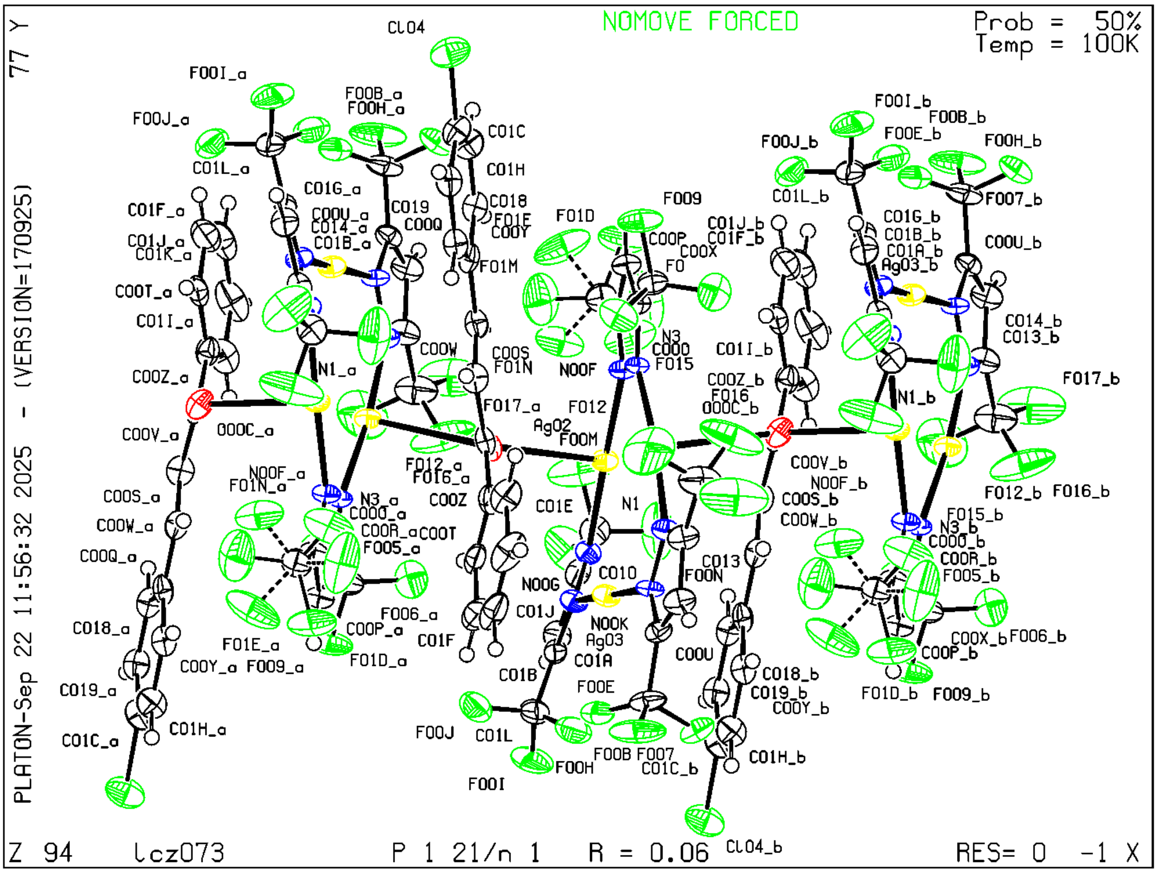

Supplement: Supplementary file 2 — Supporting File 2: anie72462‐sup‐0002‐DataFile.zip. [file ANIE-65-e1218503-s002.zip › supplementary_materials_crystallographic_data submit.zip!/cif_and_checkcif(1-114)/Ag3Pz3_73/Ag3Pz3_73_checkcif.pdf]
